# Supplementary material for: Sustained Low Incidence of Severe and Fatal COVID-19 Following Widespread Infection Induced Immunity after the Omicron (BA.1) Dominant in Gauteng, South Africa: An Observational Study
Source: Viruses. 2023 Feb 21;15(3):597. doi: 10.3390/v15030597 (PMC10054630; doi:10.3390/v15030597)
Supplement: Supplementary file 1 [file viruses-15-00597-s001.zip › viruses-2187825-supplementary.pdf]

Supplementary Material

Contents	Page
**Supplementary text**	**2**
Study setting and sero-survey	2
COVID-19 vaccine doses	2
Study limitations	2
**Supplementary Tables**	
Table S1: Time period over which sero-survey was undertaken in different sub-districts in Gauteng, South Africa.	3
Table S2a: Vaccination classification of individuals with paired samples for immunoglobulin G analysis at the pre-BA.1 and post-BA.1 sero-surveys.	4
Table S2b: Criteria used to determine seroconversion or seroresponse in the pre-Omicron and post Omicron BA.1 sero-survey interval period	4
Table S3: Demographic characteristics and anti-nucleocapsid or anti-spike protein IgG seroprevalence in individuals sampled in only post-BA.1 sero-survey and those samples in both pre-BA.1 and post-BA.1 sero-surveys.	5
Table S4: Seroprevalence of SARS-CoV-2 anti-spike (anti-S) or anti-nucleocapsid (anti-N) immunoglobulin G (IgG) Gauteng Province across the Districts and sub-Districts irrespective of Covid-19 vaccination status.	6
Table S5: Seroprevalence of SARS-CoV-2 anti-spike (anti-S) or anti-nucleocapsid (anti-N) immunoglobulin G (IgG) Gauteng Province across the Districts and sub-Districts vaccinated individuals older than 12 years only.	7
Table S6: Seroprevalence of SARS-CoV-2 anti-spike (anti-S) or anti-nucleocapsid (anti-N) immunoglobulin G (IgG) Gauteng Province across the Districts and sub-Districts unvaccinated individuals older than 12 years only.	7
Table S7: Sero-response to SARS-CoV-2 anti-spike (anti-S) and anti-nucleocapsid (anti-N) immunoglobulin G (IgG) in paired samples from individuals who did not receive any Covid-19 vaccine following the pre-BA.1 sero-survey stratified by district, age-groups and vaccination status at pre-BA.1 sero-survey.	8
Table S8: Seroconversion, sero-response and overall serological evidence of SARS-CoV-2 infection between the pre-BA.1 and post-BA.1 sero-surveys in individuals who did not receive any Covid-19 vaccine following the pre-BA.1 sero-survey and stratified by vaccination status at pre-Omicron sero-survey.	9
Table S9: Seroconversion, sero-response and overall serological evidence of SARS-CoV-2 infection between the pre-BA.1 and post-BA.1 sero-surveys in individuals who received any Covid-19 vaccine following the pre-BA.1 sero-survey and stratified by vaccination status at pre-BA.1 sero-survey.	10
Table S10: Cumulative reported Covid-19 cases, hospitalizations, recorded deaths, and excess mortality in Gauteng Province by age-group and Covid-19 wave	10
**Supplementary Figures**	
Figure S1: Seroprevalence in pre- and post-Omicron serosurvey in unvaccinated individuals	13
**Supplementary References**	13

SUPPLEMENTARY METHODS

Study setting and sero-survey

Gauteng is home to 26% (n=15.5 million) of South Africa's population. The sero-survey included households involved in the two earlier surveys. Nevertheless, based on availability and willingness to participate, the study design allowed for replacement households and different individuals within the same household which was previously involved to participate in the separate surveys. Details of the sampling framework, data collection tools and sample size calculation to enable adequate power to evaluate sero-positivity for SARS-CoV-2 at the district and sub-district levels has been previously described [1]. As done with the preceding survey, there was a 10% increase in the households that were sampled in each of the pre-defined clusters to account for possible nonparticipation, out-migration, and death since the previous survey. The earlier SARS-CoV-2 seroepidemilogical survey was conducted between October 22 to December 9, 2021. The seroprevalence of SARS-CoV-2 IgG ranged from 56.2% (95% confidence interval [CI], 52.6 to 59.7) among children younger than 12 years of age to 79.7% (95% CI, 77.6 to 81.5) among adults older than 50 years of age. At the time, only 18.8% (1318/7010) of sampled participants had received at least a single dose of Covid-19 vaccine, and they were more likely to be seropositive (93.1%) for SARS-CoV-2 than unvaccinated participants (68.4%). We also reported at the time that the incidence of infection was decoupled from the incidences of hospitalization, recorded death, and excess death during the Omicron BA.1 dominant wave, as compared with the proportions seen during previous waves.

COVID-19 vaccine doses

Of the 7,010 participants in the second sero-survey, 1,322 (18.9) had previously been vaccinated (1,025 with vaccination card seen by interviewer). 572 (43.3%) with one dose, 744 (56.3%) with two doses and 6 (0.5%) with three doses of a covid vaccine.

Of the 7,510 participants in the third sero-survey, 1,995 (26.6%) had previously been vaccinated (1,302 with vaccination card seen by interviewer). 784 (39.3%) with one dose, 1,132 (56.7%) with two doses and 79 (4.0%) with three doses of a covid vaccine.

DISCUSSION

Study limitations:

Limitations of our study include that we were unable to adequately infer the number of SARS-CoV-2 infections during the first three Covid-19 waves, as the use of a single cross-sectional survey pre-BA.1 dominant would have not accounted for re-infections which may have transpired. Although we did an earlier survey at the time of the peak of the second wave due to the Beta VOC, we used a different anti-RBD assay which was subsequently shown not to be as sensitive as the composite of the anti-N and anti-S assays in detecting previous infections. Hence, we may have over-estimated the infection to case ratios in the pre-BA.1 dominant era, as well as possibly the IFR. It is possible that individuals who were anti-N and anti-IgG negative at the pre-BA.1 survey may have been previously infected but were sero-negative due to waning of IgG to un-detectable levels, hence the group should not be regarded as being SARS-CoV-2 naïve at any stage in our survey. Also, our study is unable to adjust for different interventions which have undergone continuous but variable changes during the course of the pandemic, which could have affected susceptibility to infections, as well as progression to severe disease. Nevertheless, South Africa response to the BA.1 dominant wave was to remain at the lowest level of restrictions of activity in society, which has remained the case even with the BA.4/BA.5 dominant wave. All the regulations aimed at limiting transmission of SARS-CoV-2 were formally terminated by Government on June 22, 2022; after the BA.4/BA.5 dominant wave had subsided. Also, access to health care, including hospitalization has been less of an issue during the course of the BA.1 and BA.4/BA.5 dominant wave compared with the earlier waves when health care facilities were overwhelmed which could have resulted in higher case fatality risks in earlier waves. The extreme infectiousness of Omicron and its

sub-lineages could have resulted in significant misclassification of Covid-19 hospitalisation and reported death data, as the DATACov surveillance as it has not been possible to distinguish between those admitted or dying with SARS-CoV-2 infection, as opposed to dying from Covid-19. Consequently, the recorded Covid-19 hospitalizations and deaths during the BA.1- and BA.4/BA.5-dominant waves may be over-estimating the burden of severe Covid-19 during these waves compared with pre-BA.1 period. Lastly, although the surveillance systems have remained the same, it is likely that testing for SARS-CoV-2 infection has declined, especially after the BA.1- dominant wave. In all likelihood, there has been even further decline in people being forthcoming to be tested and consequently further decrease in testing rates since Covid-19 regulated restrictions were lifted in South Africa. Consequently, the sensitivity of the surveillance systems in detecting recorded SARS-CoV-2 infections has likely declined after the BA.1 wave. Nevertheless, the practice of testing for SARS-CoV-2 in individuals with suspected Covid-19 is unlikely to have changed. Consequently, the sensitivity of the surveillance for identifying recorded Covid-19 hospitalizations and deaths are unlikely to have changed, yielding reasonable estimates on the temporal changes in incidence of Covid-19 severe disease and death.

Table S1. Time period over which sero-survey was undertaken in different sub-districts in Gauteng, South Africa.

Sub-district	survey timelines	
Johannesburg A	01-Mar-22	24-Mar-22
Johannesburg B	24-Mar-22	11-Apr-22
Johannesburg C	01-Mar-22	04-Apr-22
Johannesburg D	01-Mar-22	30-Mar-22
Johannesburg E	01-Apr-22	07-Apr-22
Johannesburg F	01-Mar-22	06-Apr-22
Johannesburg G	02-Mar-22	07-Apr-22
Ekurhuleni E1	02-Mar-22	05-Apr-22
Ekurhuleni E2	02-Mar-22	25-Mar-22
Ekurhuleni N1	14-Mar-22	06-Apr-22
Ekurhuleni N2	01-Mar-22	08-Apr-22
Ekurhuleni S1	14-Mar-22	24-Mar-22
Ekurhuleni S2	01-Mar-22	28-Mar-22
Emfuleni	02-Mar-22	26-Mar-22
Lesedi	31-Mar-22	07-Apr-22
Midvaal	28-Mar-22	01-Apr-22
Tshwane Region 1	02-Mar-22	07-Apr-22
Tshwane Region 2	15-Mar-22	01-Apr-22
Tshwane Region 3	31-Mar-22	07-Apr-22
Tshwane Region 4	01-Apr-22	04-Apr-22
Tshwane Region 5	02-Mar-22	21-Mar-22
Tshwane Region 6	20-Mar-22	06-Apr-22
Tshwane Region 7	30-Mar-22	01-Apr-22
Merafong City	01-Mar-22	13-Mar-22
Mogale City	20-Mar-22	02-Apr-22
Rand West City	01-Mar-22	07-Apr-22

Table S2a: Vaccination classification of individuals with paired samples for immunoglobulin G analysis at the pre-BA.1 and post-BA.1 sero-surveys.

<table>
<tr><th rowspan="2">Covid-19 vaccination history reported in pre-Omicron and post Omicron BA.1[1]</th><th rowspan="2">Count</th><th colspan="2">Covid-19 vaccine classification</th></tr>
<tr><th>Vaccinated prior to pre-BA.1 survey</th><th>Vaccinated between pre-BA.1 and post-BA.1 survey</th></tr>
<tr><td>Not vaccinated</td><td>1316</td><td rowspan="2">No</td><td rowspan="7">No</td></tr>
<tr><td>Not vaccinated at the pre-Omicron survey and vaccination unknown at post Omicron BA.1 survey</td><td>222</td></tr>
<tr><td>Not vaccinated at the pre-Omicron survey, vaccinated at post Omicron BA.1 survey and vaccination date prior to pre-Omicron survey</td><td>54</td><td rowspan="5">Yes</td></tr>
<tr><td>Vaccinated at the pre-Omicron survey and not vaccinated at post Omicron BA.1 survey</td><td>180</td></tr>
<tr><td>Vaccinated at the pre-Omicron survey and vaccinated at post Omicron BA.1 survey with dates unknown</td><td>84</td></tr>
<tr><td>Vaccinated at the pre- Omicron survey and vaccination at post Omicron BA.1 survey unknown</td><td>3</td></tr>
<tr><td>Vaccinated at the pre-Omicron survey, vaccinated at post Omicron BA.1 survey and vaccination date prior to pre-Omicron survey</td><td>199</td></tr>
<tr><td>Not vaccinated at the pre-Omicron survey and vaccinated at post Omicron BA.1 survey with dates unknown</td><td>157</td><td rowspan="2">No</td><td rowspan="3">Yes</td></tr>
<tr><td>Not vaccinated at the pre-Omicron survey and vaccinated between pre-Omicron and post Omicron BA.1 survey</td><td>115</td></tr>
<tr><td>Vaccinated at the pre-Omicron survey and vaccinated between pre-Omicron and post Omicron BA.1 survey</td><td>90</td><td>Yes</td></tr>
</table>

[1]Vaccination was self-reported and may contain inconsistencies (for example, participant reported vaccination pre-Omicron survey and subsequently reported never vaccinated post-Omicron survey).

Table S2b: Criteria used to determine seroconversion or seroresponse in the pre-Omicron and post Omicron BA.1 sero-survey interval period.

<table>
<tr><th rowspan="2"></th><th colspan="2">Anti-N and anti-S serostatus at pre-Omicron survey</th><th rowspan="2">Anti-N or anti-S response at post Omicron BA.1 survey</th><th rowspan="2">Seroconversion or sero-response classification</th></tr>
<tr><th>Anti-N IgG</th><th>Anti-S IgG</th></tr>
<tr><td rowspan="8">Individuals not vaccinated between the pre-Omicron and post Omicron BA.1 sero-surveys</td><td>Neg</td><td>Neg</td><td>Anti-N IgG+</td><td rowspan="2">Sero-conversion</td></tr>
<tr><td>Neg</td><td>Neg</td><td>Anti-S IgG+</td></tr>
<tr><td>Neg</td><td>Pos</td><td>≥2 fold increase in anti-S IgG</td><td rowspan="6">Sero-response</td></tr>
<tr><td>Neg</td><td>Pos</td><td>Anti-N IgG+</td></tr>
<tr><td>Pos</td><td>Neg</td><td>≥2 fold increase in anti-N IgG</td></tr>
<tr><td>Pos</td><td>Neg</td><td>Anti-S IgG+</td></tr>
<tr><td>Pos</td><td>Pos</td><td>≥2 fold increase in anti-N IgG</td></tr>
<tr><td>Pos</td><td>Pos</td><td>≥2 fold increase in anti-S IgG</td></tr>
<tr><td rowspan="3">Individuals vaccinated between the pre-Omicron and post Omicron BA.1 sero-surveys</td><td>Neg</td><td>Neg</td><td>Anti-N IgG+</td><td>Sero-conversion</td></tr>
<tr><td>Neg</td><td>Pos</td><td>Anti-N IgG+</td><td rowspan="2">Sero-response</td></tr>
<tr><td>Pos</td><td>Neg</td><td>≥2 fold increase in anti-N IgG</td></tr>
</table>

	Pos	Pos	≥2 fold increase in anti-N IgG

Anti-N IgG= anti-nucleopcapsid immunoglobulin G; anti-S –anti-Spike immunoglobulin G.

Table S3. Demographic characteristics and anti-nucleocapsid or anti-spike protein IgG seroprevalence in individuals sampled in only post-BA.1 sero-survey and those samples in both pre-BA.1 and post-BA.1 sero-surveys.

**Category**	**Only sampled post-BA.1 sero-survey**		**Sampled in pre-BA.1 and post-BA.1 ssero-survey**	
	**Number sampled** *N* **(%)**	**Seroprevalence[1]** *n* **(%; 95% CI[2])**	**Number sampled** *N* **(%)**	**Seroprevalence[1]** *n* **(%; 95% CI[2])**
All participants†	5090	4594 (90.3; 89.4-91.0)	2420	2229 (92.1; 91.0-93.1)
Sex: Male	2175 (42.8%)	1900 (87.4; 85.9-88.7)	944 (39%)	826 (89.7; 87.6-91.5)
Female	2906 (57.2%)	2686 (92.4; 91.4-93.3)	1474 (61%)	1389 (93.6; 92.2-94.7)
Age group – yr‡				
<12	384 (7.6%)	319 (83.1; 79.0-86.5)	206 (8.5%)	172 (86; 80.5-90.1)
12–18	361 (7.1%)	338 (93.6; 90.6-95.7)	200 (8.3%)	185 (96.4; 92.7-98.2)
>18 to 50	3300 (64.9%)	2984 (90.4; 89.4-91.4)	1318 (54.5%)	1220 (92.8; 91.3-94.1)
>50	1039 (20.4%)	948 (91.2; 89.4-92.8)	696 (28.8%)	639 (91.3; 89.0-93.2)
Vaccination status‡				
Not vaccinated (all ages)	3395 (66.9%)	3015 (88.8; 87.7-89.8)	1658 (68.5%)	1362 (91; 89.5-92.4)
Vaccinated	1296 (25.5%)	1246 (96.1; 94.9-97.1)	556 (23%)	672 (96.1; 94.4-97.3)
<12yrs	384 (7.6%)	319 (83.1; 79.0-86.5)	206 (8.5%)	172 (86; 80.5-90.1)
Vaccination by age group				
<12 unvaccinated	384 (7.6%)	319 (83.1; 79.0-86.5)	206 (8.5%)	172 (86; 80.5-90.1)
12–18 unvaccinated	291 (5.7%)	268 (92.1; 88.4-94.7)	191 (7.9%)	144 (95.4; 90.7-97.7)
12–18 vaccinated	70 (1.4%)	70 (100; 94.8-100.0)	9 (0.4%)	36 (100; 90.4-100.0)
>18 to 50 unvaccinated	2521 (49.7%)	2239 (88.8; 87.5-90.0)	1059 (43.8%)	870 (91.7; 89.7-93.3)
>18 to 50 vaccinated	770 (15.2%)	736 (95.6; 93.9-96.8)	259 (10.7%)	346 (96.1; 93.6-97.7)
>50 unvaccinated	583 (11.5%)	508 (87.1; 84.2-89.6)	408 (16.9%)	348 (87.9; 84.3-90.7)
>50 vaccinated	456 (9%)	440 (96.5; 94.4-97.8)	288 (11.9%)	290 (95.7; 92.8-97.5)
Reported previous covid -19 positive test				
Never tested	4903 (96.6%)	4424 (90.2; 89.4-91.0)	2047 (84.6%)	2123 (92.1; 90.9-93.1)
Tested positive	31 (0.6%)	31 (100; 89.0-100.0)	69 (2.9%)	12 (100; 75.8-100.0)
Tested negative	143 (2.8%)	127 (88.8; 82.6-93.0)	304 (12.6%)	80 (93; 85.6-96.8)
Smoking status¶				
Non-smoker	2954 (58.2%)	2699 (91.4; 90.3-92.3)	1474 (60.9%)	1287 (93.1; 91.7-94.3)
Daily	920 (18.1%)	810 (88; 85.8-90.0)	369 (15.2%)	363 (88.3; 84.9-91.1)
Once or twice a week	271 (5.3%)	245 (90.4; 86.3-93.4)	107 (4.4%)	116 (95.1; 89.7-97.7)
Occasionally	185 (3.6%)	169 (91.4; 86.4-94.6)	64 (2.6%)	88 (94.6; 88.0-97.7)
<18yrs	745 (14.7%)	657 (88.2; 85.7-90.3)	406 (16.8%)	357 (91.1; 87.8-93.5)
Comorbidities				
None	3637 (71.6%)	3288 (90.4; 89.4-91.3)	1571 (64.9%)	1470 (92.1; 90.7-93.3)
1 or more	695 (13.7%)	637 (91.7; 89.4-93.5)	443 (18.3%)	388 (93.3; 90.4-95.3)
<18yrs (not assessed)	745 (14.7%)	657 (88.2; 85.7-90.3)	406 (16.8%)	357 (91.1; 87.8-93.5)
HIV status				
HIV negative	4640 (91.4%)	4188 (90.3; 89.4-91.1)	2223 (91.9%)	2044 (92.5; 91.3-93.5)
HIV positive	437 (8.6%)	394 (90.2; 87.0-92.6)	197 (8.1%)	171 (88.1; 82.8-92.0)

Note: Missing in post-Omicron BA.1 sero-survey: sex= 9; age-group =6; vaccination status =15; ever tested covid = 15, smoke=15; co-morbidities=13 and self-reported HIV=13. Missing in both pre-

Omicron and post-Omicron BA.1 sero-survey: sex= 4. [1]Seroprevalence was defined as seropositive for anti-S or anti-N IgG, irrespective of vaccinations status. [2]CI, confidence interval; Confidence intervals have not been adjusted for multiplicity and should not be used for inference.

Table S4. Seroprevalence of SARS-CoV-2 anti-spike (anti-S) or anti-nucleocapsid (anti-N) immunoglobulin G (IgG) Gauteng Province across the Districts and sub-Districts irrespective of Covid-19 vaccination status.

		Pre-BA.1 sero-survey		Post-BA.1 sero-survey	
**District / sub-district**	**Population total**	**Number sampled**	**Seroprevalence[1] *n* (%; 95% CI[2])**	**Number sampled**	**Seroprevalence[1] *n* (%; 95% CI[2])**
Anti-S or anti-Gauteng Province	15,176,113	7010	5124 (73.1; 72.0-74.1)	7510	6823 (90.9; 90.2-91.5)
Johannesburg District	5,606,238	2468	1880 (76.2; 74.5-77.8)	2630	2412 (91.7; 90.6-92.7)
Johannesburg A	779,519	333	246 (73.9; 68.9-78.3)	358	325 (90.8; 87.3-93.4)
Johannesburg B	435,241	197	169 (85.8; 80.2-90.0)	249	239 (96; 92.8-97.8)
Johannesburg C	799,980	444	363 (81.8; 77.9-85.1)	476	455 (95.6; 93.3-97.1)
Johannesburg D	1,396,243	646	472 (73.1; 69.5-76.3)	647	572 (88.4; 85.7-90.7)
Johannesburg E	601,433	161	117 (72.7; 65.3-79.0)	171	160 (93.6; 88.8-96.4)
Johannesburg F	751,484	243	185 (76.1; 70.4-81.1)	277	249 (89.9; 85.8-92.9)
Johannesburg G	842,339	444	328 (73.9; 69.6-77.7)	452	412 (91.2; 88.2-93.4)
Ekurhuleni District	3,825,650	1861	1382 (74.3; 72.2-76.2)	2132	1982 (93; 91.8-94.0)
Ekurhuleni E1	626 517	353	242 (68.6; 63.5-73.2)	374	332 (88.8; 85.2-91.6)
Ekurhuleni E2	455 262	252	190 (75.4; 69.7-80.3)	288	273 (94.8; 91.6-96.8)
Ekurhuleni N1	708 290	358	244 (68.2; 63.2-72.8)	378	344 (91; 87.7-93.5)
Ekurhuleni N2	697 175	258	206 (79.8; 74.5-84.3)	244	238 (97.5; 94.7-98.9)
Ekurhuleni S1	673 758	210	172 (81.9; 76.1-86.5)	243	229 (94.2; 90.6-96.5)
Ekurhuleni S2	664 648	430	328 (76.3; 72.0-80.1)	605	566 (93.6; 91.3-95.2)
Sedibeng District	1,084,503	564	398 (70.6; 66.7-74.2)	624	557 (89.3; 86.6-91.5)
Lesedi	127 419	408	293 (71.8; 67.3-76.0)	443	399 (90.1; 86.9-92.5)
Midvaal	126 285	104	65 (62.5; 52.9-71.2)	107	96 (89.7; 82.5-94.2)
Emfuleni	830 798	52	40 (76.9; 63.9-86.3)	74	62 (83.8; 73.8-90.5)
City of Tshwane District	3,709,635	1464	975 (66.6; 64.1-69.0)	1455	1255 (86.3; 84.4-87.9)
Tshwane 1	1 032 885	471	298 (63.3; 58.8-67.5)	470	385 (81.9; 78.2-85.1)
Tshwane 2	436 950	175	103 (58.9; 51.5-65.9)	175	144 (82.3; 76.0-87.2)
Tshwane 3	730 788	229	177 (77.3; 71.4-82.2)	137	122 (89.1; 82.7-93.3)
Tshwane 4	482 448	78	46 (59; 47.9-69.2)	97	83 (85.6; 77.2-91.2)
Tshwane 5	119 190	204	129 (63.2; 56.4-69.6)	236	212 (89.8; 85.3-93.1)
Tshwane 6	768 446	245	175 (71.4; 65.5-76.7)	254	229 (90.2; 85.9-93.2)
Tshwane 7	138 928	62	47 (75.8; 63.8-84.8)	86	80 (93; 85.6-96.8)
West Rand District	950,088	653	489 (74.9; 71.4-78.1)	669	617 (92.2; 89.9-94.0)
Mogale City	435 254	149	95 (63.8; 55.8-71.0)	162	146 (90.1; 84.6-93.8)
Rand West City	300 960	261	208 (79.7; 74.4-84.1)	261	246 (94.3; 90.7-96.5)
Merafong City	213 874	243	186 (76.5; 70.8-81.4)	246	225 (91.5; 87.3-94.3)

[1]Seroprevalence was defined as seropositive for anti-S or anti-N IgG, irrespective of vaccinations status. [2]CI, confidence interval; Confidence intervals have not been adjusted for multiplicity and should not be used for inference.

Table S5. Seroprevalence of SARS-CoV-2 anti-spike (anti-S) or anti-nucleocapsid (anti-N) immunoglobulin G (IgG) Gauteng Province across the Districts and sub-Districts vaccinated individuals older than 12 years only.

District / sub-district	Population total	Pre-BA.1 sero-survey		Post-BA.1 sero-survey	
		Number sampled	Seroprevalence[1] *n* (%; 95% CI[2])	Number sampled	Seroprevalence[1] *n* (%; 95% CI[2])
Anti-S or anti-Gauteng Province	15,176,113	1319	1228 (93.1; 91.6-94.3)	1995	1918 (96.1; 95.2-96.9)
Johannesburg District	5,606,238	422	401 (95; 92.5-96.7)	732	706 (96.4; 94.8-97.6)
Johannesburg A	779,519	38	36 (94.7; 82.7-98.5)	140	137 (97.9; 93.9-99.3)
Johannesburg B	435,241	33	32 (97; 84.7-99.5)	75	74 (98.7; 92.8-99.8)
Johannesburg C	799,980	73	71 (97.3; 90.5-99.2)	125	120 (96; 91.0-98.3)
Johannesburg D	1,396,243	113	104 (92; 85.6-95.8)	190	181 (95.3; 91.2-97.5)
Johannesburg E	601,433	26	26 (100; 87.1-100.0)	37	36 (97.3; 86.2-99.5)
Johannesburg F	751,484	31	30 (96.8; 83.8-99.4)	46	42 (91.3; 79.7-96.6)
Johannesburg G	842,339	108	102 (94.4; 88.4-97.4)	119	116 (97.5; 92.8-99.1)
Ekurhuleni District	3,825,650	393	373 (94.9; 92.3-96.7)	513	497 (96.9; 95.0-98.1)
Ekurhuleni E1	626 517	87	83 (95.4; 88.8-98.2)	143	140 (97.9; 94.0-99.3)
Ekurhuleni E2	455 262	53	51 (96.2; 87.2-99.0)	62	59 (95.2; 86.7-98.3)
Ekurhuleni N1	708 290	75	70 (93.3; 85.3-97.1)	128	124 (96.9; 92.2-98.8)
Ekurhuleni N2	697 175	32	32 (100; 89.3-100.0)	37	37 (100; 90.6-100.0)
Ekurhuleni S1	673 758	38	37 (97.4; 86.5-99.5)	23	22 (95.7; 79.0-99.2)
Ekurhuleni S2	664 648	108	100 (92.6; 86.1-96.2)	120	115 (95.8; 90.6-98.2)
Sedibeng District	1,084,503	114	102 (89.5; 82.5-93.9)	172	168 (97.7; 94.2-99.1)
Lesedi	127 419	81	74 (91.4; 83.2-95.8)	136	133 (97.8; 93.7-99.2)
Midvaal	126 285	20	15 (75; 53.1-88.8)	25	24 (96; 80.5-99.3)
Emfuleni	830 798	13	13 (100; 77.2-100.0)	11	11 (100; 74.1-100.0)
City of Tshwane District	3,709,635	232	208 (89.7; 85.1-92.9)	331	309 (93.4; 90.1-95.6)
Tshwane 1	1 032 885	94	82 (87.2; 79.0-92.5)	138	125 (90.6; 84.5-94.4)
Tshwane 2	436 950	25	22 (88; 70.0-95.8)	47	46 (97.9; 88.9-99.6)
Tshwane 3	730 788	52	49 (94.2; 84.4-98.0)	35	34 (97.1; 85.5-99.5)
Tshwane 4	482 448	17	15 (88.2; 65.7-96.7)	18	17 (94.4; 74.2-99.0)
Tshwane 5	119 190	10	9 (90; 59.6-98.2)	35	34 (97.1; 85.5-99.5)
Tshwane 6	768 446	22	20 (90.9; 72.2-97.5)	39	35 (89.7; 76.4-95.9)
Tshwane 7	138 928	12	11 (91.7; 64.6-98.5)	19	18 (94.7; 75.4-99.1)
West Rand District	950,088	158	144 (91.1; 85.7-94.6)	247	238 (96.4; 93.2-98.1)
Mogale City	435 254	33	28 (84.8; 69.1-93.3)	72	69 (95.8; 88.5-98.6)
Rand West City	300 960	59	58 (98.3; 91.0-99.7)	95	95 (100; 96.1-100.0)
Merafong City	213 874	66	58 (87.9; 77.9-93.7)	80	74 (92.5; 84.6-96.5)

[1]Seroprevalence was defined as seropositive for anti-S or anti-N IgG, irrespective of vaccinations status. [2]CI, confidence interval; Confidence intervals have not been adjusted for multiplicity and should not be used for inference.

Table S6. Seroprevalence of SARS-CoV-2 anti-spike (anti-S) or anti-nucleocapsid (anti-N) immunoglobulin G (IgG) Gauteng Province across the Districts and sub-Districts unvaccinated individuals older than 12 years only.

District / sub-district	Population total	Pre-BA.1 sero-survey		Post-BA.1 sero-survey	
		Number sampled	Seroprevalence[1] *n* (%; 95% CI[2])	Number sampled	Seroprevalence[1] *n* (%; 95% CI[2])
Anti-S or anti-N IgG Gauteng Province	15,176,113	4938	3473 (70.3; 69.0-71.6)	4891	4377 (89.5; 88.6-90.3)
Johannesburg District	5,606,238	1904	1396 (73.3; 71.3-75.3)	1785	1611 (90.3; 88.8-91.5)

Johannesburg A	779,519	283	201 (71; 65.5-76.0)	200	171 (85.5; 80.0-89.7)
Johannesburg B	435,241	164	137 (83.5; 77.1-88.4)	173	164 (94.8; 90.4-97.2)
Johannesburg C	799,980	352	280 (79.5; 75.0-83.4)	335	320 (95.5; 92.7-97.3)
Johannesburg D	1,396,243	500	350 (70; 65.8-73.9)	409	349 (85.3; 81.6-88.4)
Johannesburg E	601,433	130	88 (67.7; 59.2-75.1)	134	124 (92.5; 86.8-95.9)
Johannesburg F	751,484	197	147 (74.6; 68.1-80.2)	222	201 (90.5; 86.0-93.7)
Johannesburg G	842,339	278	193 (69.4; 63.8-74.5)	312	282 (90.4; 86.6-93.2)
Ekurhuleni District	3,825,650	1234	872 (70.7; 68.1-73.1)	1408	1294 (91.9; 90.4-93.2)
Ekurhuleni E1	626 517	230	139 (60.4; 54.0-66.5)	193	158 (81.9; 75.8-86.7)
Ekurhuleni E2	455 262	159	114 (71.7; 64.2-78.1)	199	189 (95; 91.0-97.2)
Ekurhuleni N1	708 290	240	151 (62.9; 56.6-68.8)	214	193 (90.2; 85.5-93.5)
Ekurhuleni N2	697 175	193	158 (81.9; 75.8-86.7)	192	186 (96.9; 93.4-98.6)
Ekurhuleni S1	673 758	154	121 (78.6; 71.4-84.3)	194	181 (93.3; 88.9-96.0)
Ekurhuleni S2	664 648	258	189 (73.3; 67.5-78.3)	416	387 (93; 90.2-95.1)
Sedibeng District	1,084,503	330	219 (66.4; 61.1-71.2)	382	330 (86.4; 82.6-89.5)
Lesedi	127 419	241	165 (68.5; 62.3-74.0)	259	225 (86.9; 82.2-90.5)
Midvaal	126 285	60	34 (56.7; 44.1-68.4)	72	63 (87.5; 77.9-93.3)
Emfuleni	830 798	29	20 (69; 50.8-82.7)	51	42 (82.4; 69.7-90.4)
City of Tshwane District	3,709,635	1054	691 (65.6; 62.6-68.4)	981	837 (85.3; 83.0-87.4)
Tshwane 1	1 032 885	302	188 (62.3; 56.7-67.5)	278	215 (77.3; 72.1-81.9)
Tshwane 2	436 950	109	62 (56.9; 47.5-65.8)	86	69 (80.2; 70.6-87.3)
Tshwane 3	730 788	159	116 (73; 65.6-79.3)	91	79 (86.8; 78.4-92.3)
Tshwane 4	482 448	45	25 (55.6; 41.2-69.1)	69	60 (87; 77.0-93.0)
Tshwane 5	119 190	184	116 (63; 55.9-69.7)	187	167 (89.3; 84.1-93.0)
Tshwane 6	768 446	213	153 (71.8; 65.4-77.4)	211	192 (91; 86.4-94.2)
Tshwane 7	138 928	42	31 (73.8; 58.9-84.7)	59	55 (93.2; 83.8-97.3)
West Rand District	950,088	416	295 (70.9; 66.4-75.1)	335	305 (91; 87.5-93.7)
Mogale City	435 254	87	56 (64.4; 53.9-73.6)	62	54 (87.1; 76.6-93.3)
Rand West City	300 960	181	133 (73.5; 66.6-79.4)	131	122 (93.1; 87.5-96.3)
Merafong City	213 874	148	106 (71.6; 63.9-78.3)	142	129 (90.8; 85.0-94.6)

[1]Seroprevalence was defined as seropositive for anti-S or anti-N IgG, irrespective of vaccinations status. [2]CI, confidence interval; Confidence intervals have not been adjusted for multiplicity and should not be used for inference.

Table S7. Sero-response to SARS-CoV-2 anti-spike (anti-S) and anti-nucleocapsid (anti-N) immunoglobulin G (IgG) in paired samples from individuals who did not receive any Covid-19 vaccine following the pre-BA.1 sero-survey stratified by district, age-groups and vaccination status at pre-BA.1 sero-survey.

District	Overall		Unvaccinated at pre-BA.1 sero-survey		Vaccinated at pre-BA.1 sero-survey	
	**Anti-N IgG[1]** *n*/*N* **(%; 95% CI[3])**	**Anti-S IgG[2]** *n*/*N* **(%; 95% CI[3])**	**Anti-N IgG[1]** *n*/*N* **(%; 95% CI[3])**	**Anti-S IgG[2]** *n*/*N* **(%; 95% CI[3])**	**Anti-N IgG[1]** *n*/*N* **(%; 95% CI[3])**	**Anti-S IgG[2]** *n*/*N* **(%; 95% CI[3])**
Gauteng Province	697/1548 (45; 42.6-47.5)	680/1548 (43.9; 41.5-46.4)	497/1070 (46.4; 43.5-49.4)	553/1070 (51.7; 48.7-54.7)	200/478 (41.8; 37.5-46.3)	127/478 (26.6; 22.8-30.7)
Johannesburg District	251/574 (43.7; 39.7-47.8)	254/574 (44.3; 40.2-48.3)	180/401 (44.9; 40.1-49.8)	200/401 (49.9; 45.0-54.7)	71/173 (41; 34.0-48.5)	54/173 (31.2; 24.8-38.5)
Ekurhuleni District	266/529 (50.3; 46.0-54.5)	245/529 (46.3; 42.1-50.6)	189/366 (51.6; 46.5-56.7)	210/366 (57.4; 52.3-62.3)	77/163 (47.2; 39.7-54.9)	35/163 (21.5; 15.9-28.4)
Sedibeng District	33/77 (42.9; 32.4-54.0)	31/77 (40.3; 30.0-51.4)	22/50 (44; 31.2-57.7)	23/50 (46; 33.0-59.6)	11/27 (40.7; 24.5-59.3)	8/27 (29.6; 15.9-48.5)
City of Tshwane District	106/269 (39.4; 33.8-45.4)	105/269 (39; 33.4-45.0)	84/191 (44; 37.1-51.1)	87/191 (45.5; 38.6-52.6)	22/78 (28.2; 19.4-39.0)	18/78 (23.1; 15.1-33.6)
West Rand	41/99 (41.4; 32.2-51.3)	45/99 (45.5; 36.0-55.2)	22/62 (35.5; 24.7-47.9)	33/62 (53.2; 41.0-65.1)	19/37 (51.4; 35.9-66.6)	12/37 (32.4; 19.6-48.5)

Age-group stratification						
<12 years	57/126 (45.2; 36.8-53.9)	69/126 (54.8; 46.1-63.2)	57/126 (45.2; 36.8-53.9)	69/126 (54.8; 46.1-63.2)		
12 to 17 years	67/127 (52.8; 44.1-61.2)	84/127 (66.1; 57.5-73.8)	63/120 (52.5; 43.6-61.2)	79/120 (65.8; 57.0-73.7)	4/7 (57.1; 25.0-84.2)	5/7 (71.4; 35.9-91.8)
18 to 50 years	376/836 (45; 41.6-48.4)	352/836 (42.1; 38.8-45.5)	285/619 (46; 42.2-50.0)	303/619 (48.9; 45.0-52.9)	91/217 (41.9; 35.6-48.6)	49/217 (22.6; 17.5-28.6)
>50 years	193/450 (42.9; 38.4-47.5)	170/450 (37.8; 33.4-42.3)	90/198 (45.5; 38.7-52.4)	99/198 (50; 43.1-56.9)	103/252 (40.9; 35.0-47.0)	71/252 (28.2; 23.0-34.0)

[1]Seroresponse for anti-N is defined for individuals who were seropositive to either S or N at pre-Omicron sero-survey and seroconverted to N or were seropositive to N at pre-Omicron sero-survey and had a ≥2 fold increase in anti-N titers at post-Omicron BA.1 sero-survey. [2]Seroresponse for anti-S is defined for individuals who were seropositive to either S or N at pre-Omicron sero-survey and seroconverted to S or were seropositive to S at pre-Omicron sero-survey and had a ≥2 fold increase in anti-S titers at post-Omicron BA.1 sero-survey. [3]CI, confidence interval; Confidence intervals have not been adjusted for multiplicity and should not be used for inference. *The criteria used for determining seroresponse is outlined in Supplementary Table S2b.

Table S8. Seroconversion, sero-response and overall serological evidence of SARS-CoV-2 infection between the pre-BA.1 and post-BA.1 sero-surveys in individuals who did not receive any Covid-19 vaccine following the pre-BA.1 sero-survey and stratified by vaccination status at pre-Omicron sero-survey.

	**Unvaccinated at pre-BA.1 sero-survey**			**Vaccinated at pre-BA.1 sero-survey**		
**District**	**Seroconversion[1]** ***n*/ *N* (%; 95% CI[4])**	**Seroresponse for anti-N and/or anti-S IgG[2]** ***n*/ *N* (%; 95% CI[4])**	**Overall serological evidence SARS-CoV-2 infection[3]** ***n*/*N* (%; 95% CI[4])**	**Seroconversion[1]** ***n*/ *N* (%; 95% CI[4])**	**Seroresponse for anti-N and/or anti-S IgG[2]** ***n*/ *N* (%; 95% CI[4])**	**Overall serological evidence SARS-CoV-2 infection[3]** ***n*/*N* (%; 95% CI[4])**
Gauteng Province	349/468 (74.6; 70.4-78.3)	681/1070 (63.6; 60.7-66.5)	1030/1538 (67.0; 64.6-69.3)	33/42 (78.6; 64.1-88.3)	252/478 (52.7; 48.2-57.2)	285/520 (54.8; 50.5-59.0)
Johannesburg District	116/144 (80.6; 73.3-86.2)	254/401 (63.3; 58.5-67.9)	370/545 (67.9; 63.9-71.7)	8/10 (80; 49.0-94.3)	97/173 (56.1; 48.6-63.3)	105/183 (57.4; 50.1-64.3)
Ekurhuleni District	124/157 (79; 72.0-84.6)	255/366 (69.7; 64.8-74.2)	379/523 (72.5; 68.5-76.1)	9/10 (90; 59.6-98.2)	89/163 (54.6; 46.9-62.1)	98/173 (56.6; 49.2-63.8)
Sedibeng District	20/28 (71.4; 52.9-84.7)	31/50 (62; 48.2-74.1)	51/78 (65.4; 54.3-75.0)	1/2 (50; 9.5-90.5)	13/27 (48.1; 30.7-66.0)	14/29 (48.3; 31.4-65.6)
City of Tshwane District	61/101 (60.4; 50.6-69.4)	107/191 (56; 48.9-62.9)	168/292 (57.5; 51.8-63.1)	11/16 (68.8; 44.4-85.8)	30/78 (38.5; 28.4-49.6)	41/94 (43.6; 34.0-53.7)
West Rand	28/38 (73.7; 58.0-85.0)	34/62 (54.8; 42.5-66.6)	62/100 (62; 52.2-70.9)	4/4 (100; 51.0-100.0)	23/37 (62.2; 46.1-75.9)	27/41 (65.9; 50.5-78.4)
Age-group stratification						
<12 years	53/74 (71.6; 60.5-80.6)	82/126 (65.1; 56.4-72.8)	135/200 (67.5; 60.7-73.6)			
12 to 17 years	30/33 (90.9; 76.4-96.9)	88/120 (73.3; 64.8-80.4)	118/153 (77.1; 69.9-83.1)	-	6/7 (85.7; 48.7-97.4)	6/7 (85.7; 48.7-97.4)
18 to 50 years	191/250 (76.4; 70.8-81.2)	382/619 (61.7; 57.8-65.5)	573/869 (65.9; 62.7-69.0)	19/20 (95; 76.4-99.1)	113/217 (52.1; 45.4-58.6)	132/237 (55.7; 49.3-61.9)
>50 years	71/106 (67; 57.6-75.2)	125/198 (63.1; 56.2-69.5)	196/304 (64.5; 58.9-69.6)	14/22 (63.6; 43.0-80.3)	131/252 (52; 45.8-58.1)	145/274 (52.9; 47.0-58.7)

[1]Seroconversion is defined for individuals who were seronegative to both S and N at pre-Omicron sero-survey and seroconverted to either S or N at post-Omicron BA.1 sero-survey. [2]Seroresponse for anti-N and/or anti-S IgG is defined for individuals who were seropositive to either S or N at pre-Omicron sero-survey and either seroconverted to S, seroconverted to N, were seropositive to N at pre-Omicron sero-survey and had a ≥2 fold increase in anti-N titers at post-Omicron BA.1 sero-survey, or were seropositive to S at pre-Omicron sero-survey and had a ≥2 fold increase in anti-S titers at post-Omicron BA.1 sero-survey. [3]Overall serological evidence of SARS-CoV-2 infection

between in the period between the two surveys when the BA.1 wave occurred was defined as either seroconversion or sero-response for anti-N and/or anti-S IgG. [4]CI, confidence interval; Confidence intervals have not been adjusted for multiplicity and should not be used for inference.*The criteria used for determining seroconversion and sero-response is outlined in Supplementary Table S2b. .

Table S9. Seroconversion, sero-response and overall serological evidence of SARS-CoV-2 infection between the pre-BA.1 and post-BA.1 sero-surveys in individuals who received any Covid-19 vaccine following the pre-BA.1 sero-survey and stratified by vaccination status at pre-BA.1 sero-survey.

	**Unvaccinated at pre-BA.1 sero-survey**			**Vaccinated at pre-BA.1 sero-survey**		
**District**	**Seroconversion[1]** ***n/ N*** **(%; 95% CI[4])**	**Seroresponse for anti-N[2]** ***n/ N*** **(%; 95% CI[4])**	**Overall serological evidence SARS-CoV-2 infection[3]** ***n/N*** **(%; 95% CI[4])**	**Seroconversion[1]** ***n/ N*** **(%; 95% CI[4])**	**Seroresponse for anti-N[2]** ***n/ N*** **(%; 95% CI[4])**	**Overall serological evidence SARS-CoV-2 infection[4]** ***n/N*** **(%; 95% CI[5])**
Gauteng Province	51/71 (71.8; 60.5-81.0)	81/201 (40.3; 33.8-47.2)	132/272 (48.5; 42.7-54.4)	3/5 (60; 23.1-88.2)	25/85 (29.4; 20.8-39.8)	28/90 (31.1; 22.5-41.3)
Johannesburg District	20/25 (80; 60.9-91.1)	51/113 (45.1; 36.3-54.3)	71/138 (51.4; 43.2-59.6)	0/1 (0; 0.0-79.3)	7/25 (28; 14.3-47.6)	7/26 (26.9; 13.7-46.1)
Ekurhuleni District	12/20 (60; 38.7-78.1)	15/45 (33.3; 21.4-47.9)	27/65 (41.5; 30.4-53.7)	1/1 (100; 20.7-100.0)	4/23 (17.4; 7.0-37.1)	5/24 (20.8; 9.2-40.5)
Sedibeng District	2/2 (100; 34.2-100.0)	3/8 (37.5; 13.7-69.4)	5/10 (50; 23.7-76.3)	0/1 (0; 0.0-79.3)	3/8 (37.5; 13.7-69.4)	3/9 (33.3; 12.1-64.6)
City of Tshwane District	8/14 (57.1; 32.6-78.6)	7/18 (38.9; 20.3-61.4)	15/32 (46.9; 30.9-63.6)	-	5/13 (38.5; 17.7-64.5)	5/13 (38.5; 17.7-64.5)
West Rand	9/10 (90; 59.6-98.2)	5/17 (29.4; 13.3-53.1)	14/27 (51.9; 34.0-69.3)	2/2 (100; 34.2-100.0)	6/16 (37.5; 18.5-61.4)	8/18 (44.4; 24.6-66.3)
Age-group stratification						
12 to 17 years	10/13 (76.9; 49.7-91.8)	5/18 (27.8; 12.5-50.9)	15/31 (48.4; 32.0-65.2)	1/1 (100; 20.7-100.0)	-	1/1 (100; 20.7-100.0)
18 to 50 years	34/47 (72.3; 58.2-83.1)	50/118 (42.4; 33.8-51.4)	84/165 (50.9; 43.3-58.4)	0/1 (0; 0.0-79.3)	10/42 (23.8; 13.5-38.5)	10/43 (23.3; 13.2-37.7)
>50 years	7/11 (63.6; 35.4-84.8)	26/65 (40; 29.0-52.1)	33/76 (43.4; 32.9-54.6)	2/3 (66.7; 20.8-93.9)	15/43 (34.9; 22.4-49.8)	17/46 (37; 24.5-51.4)

[1]Seroconversion is defined for individuals who were seronegative to both S and N at pre-Omicron sero-survey and seroconverted to N at post-Omicron BA.1 sero-survey. [2]Seroresponse for anti-N is defined for individuals who were seropositive to either S or N at pre-Omicron sero-survey and seroconverted to N or were seropositive to N at pre-Omicron sero-survey and had a ≥2 fold increase in anti-N titers at post-Omicron BA.1 sero-survey. [3]Overall serological evidence of SARS-CoV-2 infection between in the period between the two surveys when the BA.1 wave occurred was defined as either seroconversion or seroresponse for anti-N. [4]CI, confidence interval; Confidence intervals have not been adjusted for multiplicity and should not be used for inference. *The criteria used for determining seroconversion and seroresponse is outlined in Supplementary Table S2b.

Table S10. Cumulative reported Covid-19 cases, hospitalizations and recorded deaths in Gauteng Province by age-group and Covid-19 wave.

**Outcomes**	**Pre-BA.1 period cumulative**	**BA.1 dominant wave**	**Omicron sub-lineages era**	**TOTAL**
Period of case wave	March 7, 2020 to Oct. 22, 2021	Oct.23, 2021 to March 21, 2022	March 22 to November 17, 2022	
Less than 12 years: Inferred number of infections[1]	1,712,830	2,057,225	Not applicable	
Recorded cases -no†	33865	20429	10887	65181
Cumulative case rate per 100,000 population	1111.2	670.3	357.2	2138.7
Proportion of total cumulative cases, %	52.0	31.3	16.7	100
Inferred infections: Recorded cases (95%CI)	51 (47-54)	101 (91-110)	Not applicable	

Hospitalizations– no.‡	3701	2802	1999	8502
Cumulative hospitalisation rate per 100,000 population	121.4	91.9	65.6	279
Proportion of total cumulative cases, %	43.5	33.0	23.5	100
Inferred infections: Recorded Covid-19 hospitalizations ratio (95%CI)	463 (433-492)	734 (660-801)	Not applicable	
Recorded deaths in wave – no.	127	36	20	183
Cumulative recorded death rate per 100,000 population	4.2	1.2	0.7	6.0
Proportion of total cumulative cases, %	69.4	19,7	10.9	100
Inferred infections: recorded Covid-19 deaths ratio (95%CI)	13,487 (12,623-14,327)	57,145 (51,388-62,309)	Not applicable	
Infection Fatality Risk, %	0.007	0.002		
12-17 years age: : Inferred number of infections[1]	955,108	1,005,863	Not applicable	
Recorded cases -no†	40,636	19,177	7486	67299
Cumulative case rate per 100,000 population	3114.8	1469.9	573.8	5158.5
Proportion of total cumulative cases, %	60.4	28.5	11.1	100
Inferred infections: Recorded cases ratio (95%CI)	24 (22-25)	53 (48-57)	Not applicable	
Hospitalizations– no.‡	1394	771	322	2487
Cumulative hospitalisation rate per 100,000 population	106.9	59.1	24.7	190.6
Proportion of total cumulative cases, %	56.1	31.0	12.9	100
Inferred infections: Recorded Covid-19 Hospitalization ratio (95%CI)	685(652-716)	1305 (1183-1406)	Not applicable	
Recorded deaths in wave – no.	47	14	9	70
Cumulative recorded death rate per 100,000 population	3.6	1.1	0.7	5.4
Proportion of total cumulative cases, %	67.1	20.0	12.9	100
Inferred infections: recorded Covid-19 deaths ratio (95%CI)	20,321 (19,330-21,230)	71,847 (65,138-77,439)	Not applicable	
Infection Fatality Risk, %	0.005	0.001		
18-50 years age: : Inferred number of infections[1]	4,824,076	5,881,915	Not applicable	
Recorded cases -no†	596,281	182,963	78318	857561
Cumulative case rate per 100,000 population	6680.6	2049.9	877.5	9608.0
Proportion of total cumulative cases, %	69.5	21.3	9,1	100
Inferred infections: Recorded cases ratio(95%CI)	26 (26-27)	10 (9-10)	Not applicable	
Hospitalizations– no.‡	51009	11051	4384	66444
Cumulative hospitalisation rate per 100,000 population	571.5	123.8	49.1	744.4
Proportion of total cumulative cases, %	76.8	16.6	6.6	100
Inferred infections: Recorded Covid-19 Hospitalization ratio (95%CI)	95 (93-96)	532 (506-557)	Not applicable	
Recorded deaths in wave – no.	5602	512	285	6399
Cumulative recorded death rate per 100,000 population	62.8	5.7	3.2	71.7
Proportion of total cumulative cases, %	87.5	8.0	4.5	100
Inferred infections: recorded Covid-19 deaths ratio (95%CI)	9422 (9243-9588)	1050 (999-1099)	Not applicable	
Infection Fatality Risk, %	0.01	0.10		
>50 years age: Inferred number of infections[1]	838,649	1,633,469	Not applicable	
Recorded cases -no†	255226	57173	38572	350971
Cumulative case rate per 100,000 population	10078.0	2257.6	1523.1	13858.6
Proportion of total cumulative cases, %	72.7	16.3	11.0	100
Inferred infections: Recorded cases ratio (95%CI)	3.3 (3.2-3.4)	29 (26-31)	Not applicable	
Hospitalizations– no.‡	71122	7660	5056	83838

Cumulative hospitalisation rate per 100,000 population	2808.4	302.5	199.6	3310.5
Proportion of total cumulative cases, %	84.8	9.1	6.0	100
Inferred infections: Recorded Covid-19 Hospitalization ratio (95%CI)	11.8 (11.5-12.1)	213 (195-230)	Not applicable	
Recorded deaths in wave – no.	22183	1208	668	24059
Cumulative recorded death rate per 100,000 population	875.9	47.7	26.4	950.0
Proportion of total cumulative cases, %	92.2	5.0	2.8	100
Inferred infections: recorded Covid-19 deaths ratio (95%CI)	38 (37-39)	1352 (1235-1459)	Not applicable	
Infection Fatality Risk, %	2.63	0.07		

For Covid-19 cases the waves periods for Pre-omicron cumulative, Omicron BA.1 Wave and BA4/5 resurgence were March 3, 2020 to Nov. 10, 2021; Nov. 11, 2021 – April 9, 2022 and April 10, 2022 – June 5, 2022, respectively. For Covid-19 hospitalizations the wave periods for Pre-omicron cumulative, Omicron BA.1 Wave and BA4/5 resurgence were March 7, 2020 to Nov. 21, 2021; Nov. 22, 2021 to April 12, 2022 and April 13, 2022 to June 5, 2022, respectively. For Covid-19 recorded deaths the wave periods for Pre-omicron cumulative, Omicron BA.1 Wave and BA4/5 resurgence were March 31, 2020 to Nov. 23, 2021; Nov. 24, 2021 to May 4, 2022 and May 5, 2022 - June 5, 2022, respectively.[1]The inferred number of infections in the population pre-Omicron was derived by multiplying the seroprevalence in unvaccinated individuals at the time of the pre-Omicron sero-survey by the STATS-SA population [13]. For wave 3 inferred number of infections was obtained by multiplying the proportion of unvaccinated individuals showing evidence of overall serological evidence of SARS-CoV-2 infection (Table S8) between the pre-Omicron and post-Omicron sero-surveys, by the STATS-SA population. [2] The Infection Fatality Ratio was calculated as the inverse of the Inferred Infection:recorded deaths or excess ratios.

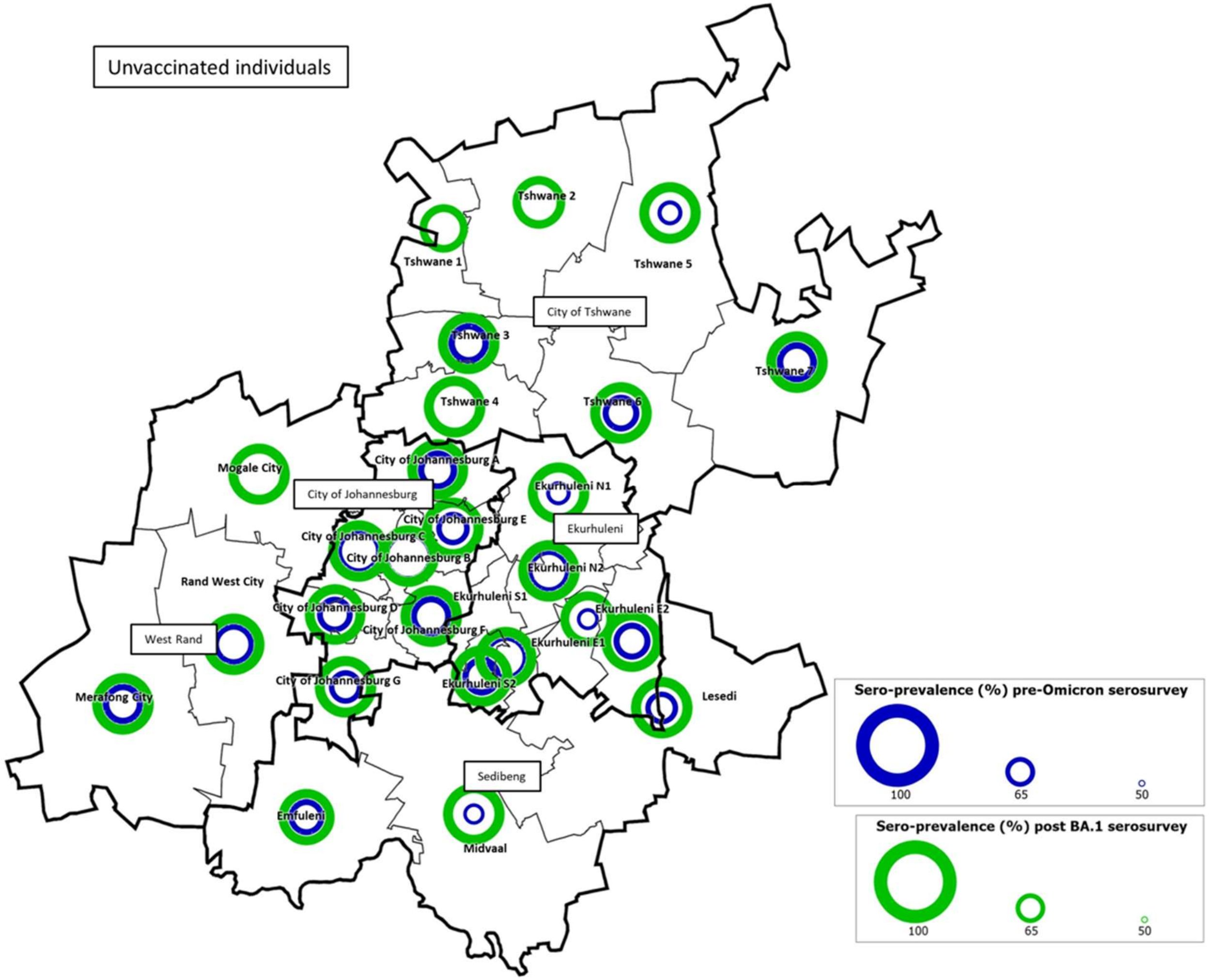

Figure S1. Seroprevalence in pre- and post-Omicron serosurvey in unvaccinated individuals.

We illustrate the change in seroprevalence pre- and post-Omicron in unvaccinated individuals across the 26 subdistricts in Gauteng province.

REFERENCES

1. Madhi SA, Kwatra G, Myers JE, et al. Population Immunity and Covid-19 Severity with Omicron Variant in South Africa. *New Eng J Med* **2022**;386:1314-26.
